# Incidence of neonatal mortality and its predictors among live births in Ethiopia: Gompertz gamma shared frailty model

**DOI:** 10.1186/s13052-020-00893-6

**Published:** 2020-09-21

**Authors:** Zemenu Tadesse Tessema, Getayeneh Antehunegn Tesema

**Affiliations:** Department of Epidemiology and Biostatistics, Institute of Public Health, College of Medicine and Health Sciences, University of Gondar, Gondar, Ethiopia

**Keywords:** Ethiopia, Gompertz gamma shared frailty, Neonatal mortality

## Abstract

**Background:**

Neonatal mortality remains a serious public health concern in developing countries including Ethiopia. Ethiopia is one of the countries with the highest neonatal mortality in Africa. However, there is limited evidence on the incidence and predictors of neonatal mortality at the national level. Therefore, this study aimed to investigate the incidence of neonatal mortality and its predictors among live births in Ethiopia. Investigating the incidence and predictors of neonatal mortality is essential to design targeted public health interventions to reduce neonatal mortality.

**Methods:**

A secondary data analysis was conducted based on the 2016 Ethiopian Demographic and Health Survey (EDHS) data. A total weighted sample of 11,022 live births was included in the analysis. The shared frailty model was applied since the EDHS data has hierarchical nature, and neonates are nested within-cluster, and this could violate the independent and equal variance assumption. For checking the proportional hazard assumption, Schoenfeld residual test was applied. Akakie Information Criteria (AIC), Cox-Snell residual test, and deviance were used for checking model adequacy and for model comparison. Gompertz gamma shared frailty model was the best-fitted model for this data since it had the lowest deviance, AIC value, and the Cox-Snell residual graph closet to the bisector. Variables with a *p*-value of less than 0.2 were considered for the multivariable Gompertz gamma shared frailty model. In the multivariable Gompertez gamma shared frailty model, the Adjusted Hazard Ratio (AHR) with a 95% confidence interval (CI) was reported to identify significant predictors of neonatal mortality.

**Results:**

Overall, the neonatal mortality rate in Ethiopia was 29.1 (95% CI: 26.1, 32.4) per 1000 live births. In the multivariable Gompertz gamma shared frailty model; male sex (AHR = 1.92, 95% CI: 1.52, 2.43), twin birth (AHR = 5.22, 95% CI: 3.62, 7.53), preceding birth interval less than 18 months (AHR = 2.07, 95% CI: 1.51, 2.85), small size at birth (AHR = 1.64, 95% CI: 1.24, 2.16), large size at birth (AHR = 1.53, 95% CI: 1.16, 2.01) and did not have Antenatal Care (ANC) visit (AHR = 2.10, 95% CI: 1.44, 3.06) were the significant predictors of neonatal mortality.

**Conclusion:**

Our study found that neonatal mortality remains a public health problem in Ethiopia. Shorter birth interval, small and large size at birth, ANC visits, male sex, and twin births were significant predictors of neonatal mortality. These results suggest that public health programs that increase antenatal care service utilization should be designed to reduce neonatal mortality and special attention should be given for twin births, large and low birth weight babies. Besides, providing family planning services for mothers to increase birth intervals and improving accessibility and utilization of maternal health care services such as ANC is crucial to improve neonatal survival.

## Background

According to the World Health Organization (WHO), neonatal mortality is defined as the death of babies within 28 days of birth [1] and is considered as the most sensitive indicators of the socioeconomic status of the community, and health care service availability and accessibility of the country [2, 3]. Globally, 2.5 million newborn babies died annually, of which 99% occurred in developing countries [4]. Neonatal mortality constitutes 40% of total under-5 mortality and 57% of global infant mortality [5]. Reducing neonatal mortality is an integral component of Goal 3 of Sustainable Development Goal (SDG) to end preventable death [6]. Globally, neonatal mortality is an unresolved Millennium Development Goals (MDG4) agenda and remains unacceptably high, with 7000 deaths a day [7].

Neonatal mortality has decreased steadily over the past two decades, although this was the slowest in Sub-Saharan Africa (SSA), where 38% of global neonatal mortality occurred [8]. Ethiopia has among African countries with the highest newborn mortality rates [9, 10]. The Neonatal Mortality Rate (NMR) in Ethiopia has decreased in the last 16 years, from 49 to 29 per 1000 live births. Still, it is far below the United Nations’ (UN) ambitious target of preventing preventable newborn deaths and reducing neonatal mortality to 12 per 1000 live births in every country by 2030 [11]. The majority of neonatal death are caused by preventable and treatable causes such as diarrheal disease, pneumonia, sepsis, asphyxia, and prematurity that can be avoided by using basic maternal and child health care services [12–14].

While Ethiopia has made large-scale investments and programs such as the implementation of the Health Extension Program (HEP), quality and accessibility of maternal healthcare services, and Millennium Development Goals, it is among the few countries with the highest NMR burden [15, 16]. Previous studies documented on neonatal mortality found that maternal age [17], residence [18], birth interval [19], birth weight [20], place of delivery [21], birth order [22], husband education [23], Antenatal Care (ANC) visit [24, 25], media exposure [25], Postnatal Care (PNC) visit [26], multiple gestation [27], and maternal education [28] were significant predictors of neonatal mortality.

According to the EDHS reports, the neonatal mortality rate decreased from 49 per 1000 births in 2000 [29] to 29 per 1000 live births in 2016 [30], which accounts for 43.3% of deaths under 5 in Ethiopia [31]. However, there is limited evidence on the incidence and predictors of neonatal mortality at the national level using survival analysis. Understanding the incidence and predictors of neonatal mortality is critical to design effective public health programs and interventions to reduce neonatal mortality in Ethiopia. Therefore, this study aimed to investigate the incidence of and predictors of neonatal mortality in Ethiopia.

## Methods

### Data source

A secondary data analysis was conducted based on the EDHS 2016 data. The 2016 EDHS survey was the fourth survey conducted in Ethiopia, situated in the Horn of Africa. Ethiopia has 9 regional states (Afar, Amhara, Benishangul-Gumuz, Gambela, Harari, Oromia, Somali, Southern Nations, Nationalities, and People’s Region (SNNPR) and Tigray) and two Administrative Cities (Addis Ababa and Dire-Dawa). The EDHS used a stratified two-stage cluster sampling technique selected in two stages using the 2007 Population and Housing Census (PHC) as a sampling frame. Stratification was achieved by separating each region into urban and rural areas. In total, 21 sampling strata have been created. In the first stage, 645 Enumeration Areas (EAs) (202 in the urban area) were selected with probability selection proportional to the EA size and independent selection in each sampling stratum. In the second stage, on average, 28 households were systematically selected. A total weighted sample of 11,022 live births within 5 years preceding the survey were included. The detailed sampling procedure was presented in the full EDHS 2016 report [32].

### Study variables

The outcome variable for this study was neonatal survival status categorized as being alive (coded as 0) or died (coded as 1). Neonatal mortality is defined as the death of live birth within 28 days of life. Age at death was recorded in days if the child died within 28 days of delivery. The independent variables considered for this study were categorized as socio-demographic and economic variables (residence, region, religion, maternal education, husband education, maternal occupation, sex of household head, distance to the health facility and wealth status), child-related factors (sex of neonate, type of birth, preceding birth interval and birth size), and maternal healthcare services related factors (ANC visit, place of delivery, mode of delivery, parity, birth order, wanted pregnancy, and health insurance coverage).

### Data management and analysis

The data were weighted using sampling weight, primary sampling unit, and strata before any statistical analysis to restore the representativeness of the survey and take into account the sampling design to get reliable statistical estimates. The sampling statisticians determine how many samples are needed in each stratum to get reliable estimates, in EDHS, some regions were oversampled, and some regions were under-sampled. So, to get statistics that are representative of Ethiopia, the distribution of neonate in the sample need to be weighted (mathematically adjusted) such that it resembles the true distribution in Ethiopia by using sampling weight (v005), primary sampling unit (v021) and strata (v022). The descriptive and summary statistics were conducted using STATA version 14 software. The EDHS data has a hierarchical structure, and therefore neonates are nested within a cluster/EAs. This violates the traditional regression model assumption, which is the independence of observations and equal variance across clusters. We have checked whether there was clustering or not by running the frailty model (random effect survival model). EA was used as a random effect (clustering variable). The theta parameter was used to assess whether there is significant clustering or not. The EDHS data were collected at two-level at individual and at the community level. Therefore, neonates in the same cluster are more of share similar characteristics than neonates in another cluster. The theta (frailty parameter) was significant at the null model (θ = 0.45, 95% CI: 0.22, 0.83). It showed that there was unobserved heterogeneity or shared frailty that needs to be taken into account to get a reliable estimate. Schoenfeld residual test was applied to check the Proportional Hazard (PH) assumptions, and the PH assumption was fulfilled (*p*-value> 0.05). For model selection, log-likelihood ratio test, deviance (−2LL), Akaike Information Criteria (AIC), and Cox-Snell residual plot were applied. Cox-Snell Residual test is the difference between an observed data point and a predicted or fitted value. A Cox-Snell residual considers the distribution and estimated parameters from the lifetime regression model. A model with the highest values of log-likelihood and the lowest value of AIC was the best-fitted model. Nested parametric models in generalized gamma (Exponential, Weibull, lognormal) were compared based on deviance, and non-nested models (Gompertz and log-logistic) were compared using AIC. Deviance, AIC, and Cox-Snell residual graph showed that the Gompertz gamma shared frailty model had the lowest value and the closet graph to the bisector. Therefore, the Gompertz gamma shared frailty model was the best-fitted model for the data.

Variable with a *p*-value less 0.20 in the uni-variable gamma shared frailty analysis were included in the multivariable analysis. We estimate the hazard ratio and 95% confidence interval. In the multivariable analysis, the Adjusted Hazard Ratio (AHR) with 95% Confidence Interval (CI) was used to declare significant predictors of neonatal mortality.

### Ethical consideration

Permission for data access was obtained from major demographic and health survey through an online request from http://www.dhsprogram.com. The data used for this study were publicly available with no personal identifier.

## Result

### Characteristics of the study population

A total of 11,022 live births were included in this study, of these, 5725 (51.9%) were males, and 292 (2.6%) were twin births. More than two-thirds (72.6%) of the births were delivered at home, and 89% were born to mothers lived in rural areas. About 5842 (53.0%) of the births were born to mothers aged 25–34 years, and 7284 (66.1%) were born to mothers who had no formal education. Three-fourth (75.1%) of the births were wanted, and 4836 (43.9%) were born to mothers who had 2–4 births (Table 1).

**Table 1:** Characteristics of the study population in Ethiopia, 2016

Characteristics	Category	Weighted frequency (%)
Residence	Rural	9807 (89.0)
	Urban	1215 (11.0)
Region	Tigray	716 (6.5)
	Afar	114 (1.0)
	Amhara	2072 (18.8)
	Oromia	4851 (44.0)
	Somali	508 (4.6)
	Benishangul-Gumuz	121 (1.1)
	SNNPRs	2296 (20.8)
	Gambella	27 (0.2)
	Harari	26 (0.2)
	Addis Ababa	243 (2.2)
	Dire Dawa	47 (0.4)
Maternal age	15–24	2446 (22.2)
	25–34	5842 (53.0)
	≥35	2734 (24.8)
Wealth index	Poorest	2636 (23.9)
	Poorer	2520 (22.9)
	Middle	2280 (20.7)
	Richer	1998 (18.1)
	Richest	1588 (14.4)
Maternal education status	No	7284 (66.1)
	Primary	2950 (26.8)
	Secondary and above	788 (7.1)
Husband education status	No	5003 (45.4)
	Primary	4112 (37.3)
	Secondary and above	1904 (17.3)
Maternal occupation	Not working	8034 (72.9)
	Working	2988 (27.1)
Child was wanted	No	2743 (24.9)
	Yes	8279 (75.1)
Sex of child	Male	5725 (51.9)
	Female	5297 (48.1)
Type of birth	Single	10,730 (97.4)
	Twin	292 (2.6)
Place of delivery	Home	7997 (72.6)
	Health facility	3025 (27.4)
Preceding birth interval (in months)	< 18	942 (8.6)
	18–36	4112 (37.3)
	≥ 37	5968 (54.1)
Birth size	Small	2957 (26.8)
	Average	4580 (41.6)
	Large	3485 (31.6)
Sex of household head	Female	1529 (13.9)
	Male	9493 (86.1)
ANC visit	No	6266 (56.9)
	Yes	4756 (43.1)
Parity	1	1434 (13.0)
	2–4	4836 (43.9)
	≥5	4752 (43.1)
Media exposure	No	7375 (66.9)
	Yes	3647 (33.1)
Covered by health insurance	No	10,633 (94.5)
	yes	389 (3.5)

### Neonatal mortality rate by respondents characteristics

From a total of 11,022 live births, 321 babies died within 28 days of birth. The overall neonatal mortality rate in Ethiopia was 29.1 [95% CI: 26.1, 32.4] per 1000 live births. The neonatal mortality rate has been varied across regions, ranged from 20.7 per 1000 live births in Addis Ababa to 40.8 per 1000 live births in the Somali region. Of the total 251 twin births, 14.1% died during the neonatal period. The neonatal mortality rate was highest among male children, mothers who had no ANC visit during pregnancy, shorter preceding birth intervals, and small birth sizes (Table 2).

**Table 2:** Neonatal mortality rates by respondents characteristics in Ethiopia, 2016

Characteristics	Category	Status		NMR per 1000 live births
		Event	Censured	
Residence	Rural	9527	280	28
	Urban	1174	41	34
Region	Tigray	696	19	26.9
	Afar	111	3	26.2
	Amhara	2006	66	32.1
	Oromia	4711	139	28.8
	Somali	487	21	40.8
	Benishangul-Gumuz	118	4	28.7
	SNNPRs	2236	60	26.2
	Gambella	26	1	28.0
	Harari	25	1	31.3
	Addis Ababa	239	5	20.7
	Dire Dawa	45	1	29.8
Maternal age	15–24	2387	59	24.0
	25–34	5687	155	26.6
	≥35	2628	106	38.9
Wealth index	Poorest	2584	52	19.7
	Poorer	2446	73	29.1
	Middle	2209	70	30.8
	Richer	1921	77	38.9
	Richest	1540	47	30.0
Maternal education status	No	7058	225	30.9
	Primary	2880	71	24.0
	Secondary and above	763	25	31.4
Husband education status	No	4850	152	30.5
	Primary	3998	118	28.7
	Secondary and above	1854	50	26.4
Maternal occupation	Not working	7798	236	29.4
	Working	2903	85	28.4
Child was wanted	No	2660	83	30.3
	Yes	8042	238	28.7
Sex of child	Male	5496	229	40.0
	Female	5206	92	17.4
Type of birth	Single	10,450	280	26.1
	Twin	251	41	141.0
Place of delivery	Home	7780	217	27.1
	Health facility	2921	104	34.3
Preceding birth interval (in months)	< 18	888	54	57.7
	18–36	4001	110	26.9
	≥ 37	5812	156	26.1
Birth size	Small	3362	122	35.1
	Average	4483	97	21.2
	Large	2856	101	34.2
Sex of household head	Female	1490	38	25.1
	Male	9211	282	29.7
ANC visit	No	6028	238	37.9
	Yes	4673	83	17.5
Parity	1	1422	12	8.9
	2–4	4684	151	31.4
	≥5	4596	156	32.9
Media exposure	No	7192	183	24.8
	Yes	3510	138	37.7

### Predictors of neonatal mortality

**Fig. 1:**
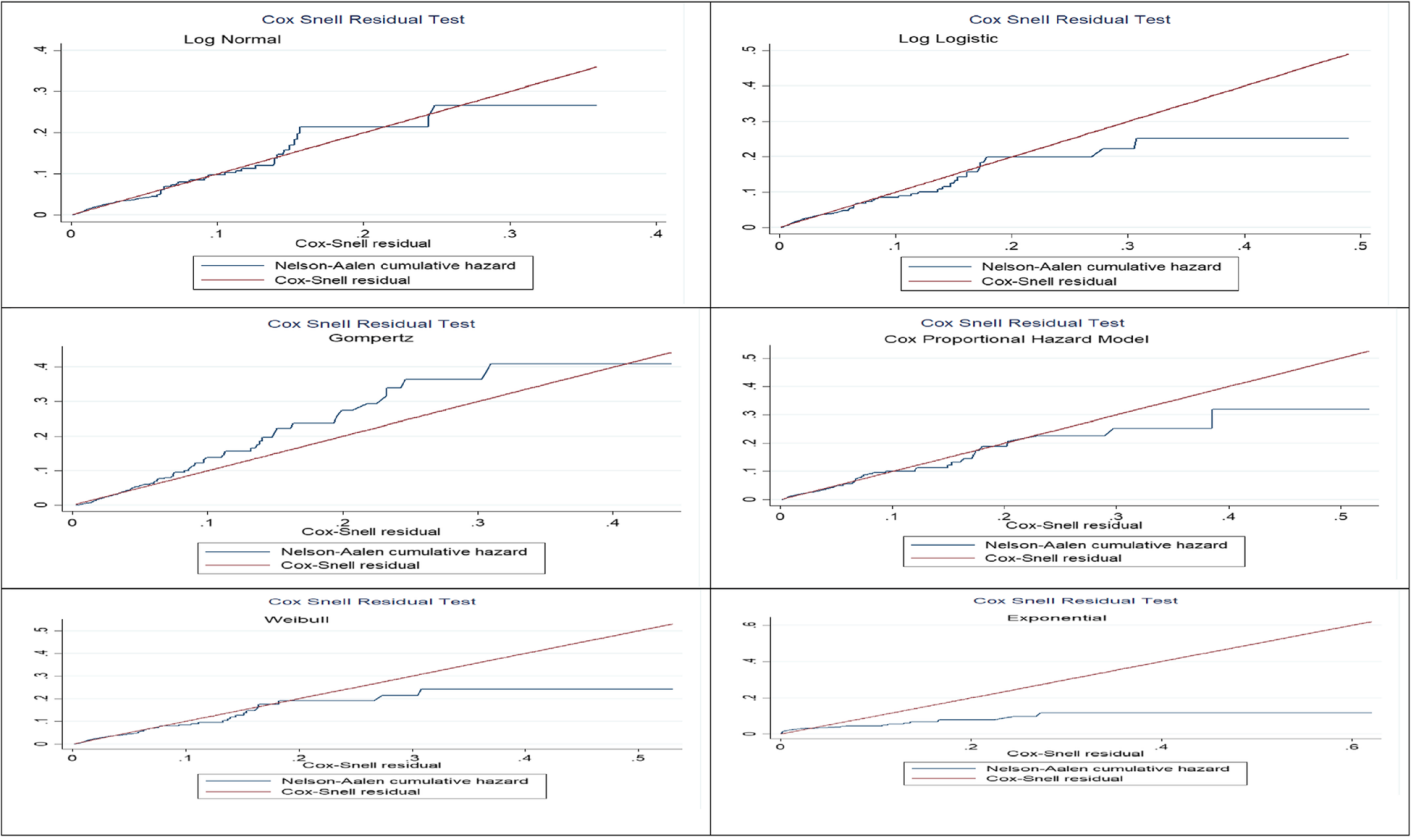
The Cox-Snell Residual test of semi parametric and parametric survival models

Based on deviance, AIC, Cox-Snell residual test, and theta value, the shared frailty model with Gompertz distribution and gamma frailty was the most efficient model for the data (Table 3, and Fig. 1). The goodness of fit for the fitted model was also performed using the Cox-Snell residual test and showed that the model was adequate.

**Table 3:** Model diagnostics and comparison

Model	Distribution	Frailty	Theta	AIC	BIC	Deviance (−2LL)
Cox proportion hazard	Unspecified	Unspecified	–	5802	5969	5756
Shared frailty	Gompertz	Gamma	0.39	3757	3946	3704
Shared frailty	Gompertz	Inverse Gaussian	0.44	3757	3946	3704
Shared frailty	Exponential	Gamma	0.51	4211	4393	4160
Shared frailty	Exponential	Inverse Gaussian	0.59	4210	4392	3850
Shared frailty	Weibull	gamma	0.40	3902	4091	3850
Shared frailty	Weibull	Inverse Gaussian	0.60	3902	4091	3850
Shared frailty	Log-normal	gamma	0.40	3878	4067	3826
Shared frailty	Log-normal	Inverse Gaussian	0.45	3910	4012	3932

In the Gompertz gamma shared frailty model; residence, maternal age, maternal education, wealth index, sex of household head, sex of neonate, type of birth, parity, preceding birth interval, birth size, media exposure, covered by health insurance, TT vaccination status and wanted birth were considered for multivariable analysis since they head *p*-value < 0.2 in the bi-variable analysis. However, in the multivariable analysis, sex of neonate, ANC visit, type of birth, preceding birth interval, and birth size were significant predictors of neonatal mortality.

The hazard of neonatal death among male births was 1.92 (AHR = 1.92, 95% CI: 1.52, 2.43) times higher than females. The hazard of neonatal death among twin births was 5.22 (AHR = 5.22, 95% CI: 3.62, 7.53) times higher than single births. Live births who were born within the preceding birth interval of fewer than 18 months was 2.07 (AHR = 2.07, 95% CI: 1.51, 2.85) times more at risk of death in the neonatal period compared to live births who had a preceding birth interval of 18–36 months. Children born to mothers who didn’t have ANC visits during pregnancy had 2.10 (AHR = 2.10, 95% CI: 1.44, 3.06) times higher risk of death during the neonatal period compared to children born to mother who had ANC visit. Those live births who were small size at birth and large size at birth was 1.64 (AHR = 1.64, 95% CI: 1.24, 2.16) and 1.53 (AHR = 1.53, 95% CI: 1.16, 2.01) times increased risk of death in the neonatal period compared to live births who were average size at birth (Table 4).

**Table 4:** Shared Gompertz distribution gamma shared frailty model for the predictors of neonatal mortality in Ethiopia, 2016

Variable	Neonate died		Crude Hazard Ratio (CHR) with 95% CI	Adjusted Hazard Ratio (AHR) with 95% CI
	No	Yes		
**Residence**				
Urban	1175	41	1	1
Rural	9527	279	1.83 [1.27, 2.64]	1.51 [0.86, 2.64]
**Maternal age**				
15–24	2387	59	0.85 [0.64, 1.15]	0.93 [0.62,1.39]
25–34	5687	155	0.66 [0.50, 0.85]	0.64 [0.48, 0.87]
≥ 35	2628	106	1	1
**Maternal education status**				
No	7058	225	1	1
Primary	2880	71	0.87 [0.66, 1.14]	1.13 [0.84, 1.52]
Secondary and higher	763	24	0.77 [0.51, 1.15]	1.36 [0.83, 2.24]
**Wealth status**				
Poorest	2584	52	1.73 [1.20, 2.50]	0.92 [0.52, 1.63]
Poorer	2446	73	1.78 [1.18, 2.68]	1.09 [0.60, 1.96]
Middle	2209	70	1.56 [1.01, 2.42]	0.99 [0.54, 1.88]
Richer	1921	78	1.49 [0.95, 2.33]	0.95 [0.53, 1.71]
Richest	1540	47	1	1
**Sex of household**				
Male	9211	282	1	1
Female	1491	38	0.65 [0.48, 0.89]	0.68 [0.49, 1.02]
**Sex of neonate**				
Female	5206	92	1	1
Male	5496	228	1.87 [1.49, 2.37]	1.92 [1.52, 2.43]^*^
**Type of birth**				
Single	10,451	279	1	1
Twin	251	41	6.12 [4.31, 8.69]	5.22 [3.62, 7.53]^*^
**Parity**				
1	1421	13	1	1
2–4	4684	151	1.73 [1.14, 2.63]	1.23 [0.77, 1.97]
≥ 5	4596	156	1.96 [1.29,2.99]	1.10 [0.63, 1.93]
**Preceding birth interval**				
18–36 months	4001	110	1	1
< 18 months	888	54	2.10 [1.53, 2.88]	2.07 [1.51, 2.85]^*^
> 36 months	5813	156	0.95 [0.75, 1.22]	1.05 [0.81, 1.38]
**Size of neonate at birth**				
Average	4483	97	1	1
Small	2856	101	1.67 [1.27, 2.19]	1.64 [1.24, 2.16]^*^
Large	3362	122	1.51 [1.15, 1.99]	1.53 [1.16, 2.01]^*^
**Media exposure**				
No	7192	183	1	1
Yes	3510	137	0.88 [0.69, 1.13]	1.14 [0.85, 1.53]
**Covered by health insurance**				
No	10,321	311	1	1
Yes	380	9	0.52 [0.21, 1.28]	0.64 [0.26, 1.59]
**ANC visit during pregnancy**				
No	6028	238	1	1
Yes	4673	83	2.95 [2.25, 3.86]	2.10 [1.44, 3.06]
**Vaccinated for TT during pregnancy**				
No	6762	249	2.67 [2.00, 3.57]	1.41[0.95, 2.10]
Yes	3940	71	1	1
**Wanted child**				
Yes	8041	237	1	1
No	2660	83	0.80 [0.59, 1.10]	0.79 [0.57, 1.09]

* = p-value < 0.01

## Discussion

The overall aim of this study was to investigate the incidence of neonatal mortality and its predictors in Ethiopia based on weighted nationally representative EDHS 2016 data using the Gompertz gamma shared frailty model. In this study, the neonatal mortality rate in Ethiopia was 29.1 (95% CI: 26.1, 32.4) per 1000 live births with an Annual Rate of Reduction (ARR) of 2.6%. This was lower than a study reported in Nigeria [33], the possible justification could be due to the difference in the study period and the difference in the study population. Besides, it could be due to the continued commitment of Ethiopia to improve maternal and newborn survival by implementation continued investment in accessing basic health care services. But this finding was higher than the United Nations Every New Born action plan [34]. This provides an insight that Ethiopia should reduce neonatal mortality to reach every born action plan by 2030.

In the Gompertz gamma shared frailty analysis; type of birth, preceding birth interval, birth size, sex of neonate, and ANC visit during pregnancy were significant predictors of neonatal mortality. In the current study, the hazard of neonatal mortality was higher among twin births than singletons. It was in line with a study reported in Ghana [35]. This might be due to the increased risk of twins to prematurity, low birth weight, Intra-Uterine Growth Restriction (IUGR), twin to twin transfusion syndrome, and congenital anomaly that increases the risk of dying during the neonatal period than singletons [36, 37]. Besides, twins are more prone to malnutrition compared to singletons, thus they are prone to infectious diseases, hypothermia, and sepsis [38] this could increase their risk of mortality during the neonatal period.

The hazard of neonatal mortality was higher among male births was than female births. This was consistent with study findings in Ethiopia [39], Sudan [40], and Pakistan [41]. The possible justification could be due to the preconception environment, thus biologically male children are at higher risk of infectious diseases due to higher prevalence of immune-deficiency, and have delayed fetal lung maturity, this could result in male neonates at higher risk of respiratory diseases and high risk of mortality [42, 43].

The preceding birth interval was a significant predictor of neonatal mortality. Having the preceding birth interval of fewer than 18 months increases neonatal mortality risk than births with preceding birth interval of 18–36 months. This is supported by previous study findings in Indonesia [19], and Nigeria [44]. The possible explanation could be due to the reason that mothers having shorter preceding birth intervals are less able to provide nourishment for the fetus because her body has less time to recuperate from the previous pregnancy. Besides, the uterus had less time to recover, and also lactation will deplete maternal nutrition [45] this could result in low birth weight, preterm labor, etc. due to lack of care, attention, and competition of children that could increase the risk of neonatal mortality.

Live births born to women who didn’t have ANC visits during pregnancy were at higher risk of death in the neonatal period than live births born to mothers who had ANC visits during pregnancy. This was consistent with study findings in Kenya [24] and Bangladesh [25]. The possible justification might be due to the reason that ANC offers pregnant women an opportunity to access preventive care, treatment, and health education, including child nutritional advice and seeking treatment [46]. Also, ANC is considered an entry point for other maternal healthcare service utilization. Women who have ANC visit are advised to seek skilled delivery and PNC visit as well as they have a good awareness about childhood illness and seeking medical care [47].

Mothers who perceived the size of their newborns were small and large size at birth had a higher risk of death in the first month of life compared to newborns who were average-sized at birth. It was consistent with a study in Bangladesh [48]. This could be because the small size and large size babies are indicators of underlying congenital abnormality including neural tube defect, congenital heart diseases, and Down syndrome and/or medical illness of the new-borns including congenital syphilis, tuberculosis, HIV cytomegalovirus, malaria, sepsis, etc. furthermore, small size babies are more prone to hypothermia, and infection, this could lead to the death of the neonate [49].

The strength of this study was, it was based on the weighted data to make it representativeness at national and regional levels. Therefore, it can be generalized to all live births during the study period in Ethiopia. Moreover, the use of shared frailty modeling that took into account the nested nature of the EDHS data and the variability within the community to get a reliable estimate and standard errors. This study had limitations. First, in EDHS, only surviving women were interviewed, and this could underestimate the neonatal mortality rate since maternal mortality is commonly associated with neonatal mortality. Furthermore, the EDHS survey did not incorporate clinically confirmed data; instead, it relied on mothers or caregivers report and might have the possibility of social desirability and recall bias, and variables like maternal obstetric complications such as Antepartum Hemorrhage (APH), preeclampsia, eclampsia, Premature Rupture of Membrane (PROM), infection, and congenital anomalies which may be considered as the most common cause of neonatal mortality were not addressed in this study because these variables were not available in EDHS data.

## Conclusions

The neonatal mortality rate in Ethiopia remains a major public health problem. Male sex, twin birth, shorter preceding birth interval, having no ANC visit, and small and large size at birth were significantly associated with increased risk of neonatal mortality. Therefore, to reduce neonatal mortality, integrated public health interventions are needed. Giving special medical care and follow up for twin births, small and large babies, and male births are mandatory to reduce the incidence of neonatal mortality. Providing family planning services for mothers to increase birth intervals and improving accessibility and utilization of maternal health care services such as ANC is crucial to improve neonatal survival.

## Data Availability

Data is available online and you can access it from www.measuredhs.com.

## Abbreviations

AHR: Adjusted Hazard Ratio
AIC: Akaike Information Criteria
ANC: Antenatal Care
BIC: Bayesian Information Criteria
CI: Confidence Interval
CHR: Crude Hazard Ratio
EAs: Enumeration Areas
EDHS: Ethiopian Demographic and Health Survey
IUGR: Intrauterine Growth Restriction
MDG: Millennium Development Goal
NMR: Neonatal Mortality Rate
SSA: Sub-Saharan Africa
WHO: World Health Organization
